# Developing an electronic health record (EHR) for methadone treatment recording and decision support

**DOI:** 10.1186/1472-6947-11-5

**Published:** 2011-02-01

**Authors:** Liang Xiao, Gráinne Cousins, Brenda Courtney, Lucy Hederman, Tom Fahey, Borislav D Dimitrov

**Affiliations:** 1 HRB Centre for Primary Care Research, Department of General Practice, Royal College of Surgeons in Ireland, Dublin, Republic of Ireland; 2 Department of Computer Science, Trinity College Dublin, Republic of Ireland

## Abstract

**Background:**

In this paper, we give an overview of methadone treatment in Ireland and outline the rationale for designing an electronic health record (EHR) with extensibility, interoperability and decision support functionality. Incorporating several international standards, a conceptual model applying a problem orientated approach in a hierarchical structure has been proposed for building the EHR.

**Methods:**

A set of archetypes has been designed in line with the current best practice and clinical guidelines which guide the information-gathering process. A web-based data entry system has been implemented, incorporating elements of the paper-based prescription form, while at the same time facilitating the decision support function.

**Results:**

The use of archetypes was found to capture the ever changing requirements in the healthcare domain and externalises them in constrained data structures. The solution is extensible enabling the EHR to cover medicine management in general as per the programme of the HRB Centre for Primary Care Research.

**Conclusions:**

The data collected via this Irish system can be aggregated into a larger dataset, if necessary, for analysis and evidence-gathering, since we adopted the openEHR standard. It will be later extended to include the functionalities of prescribing drugs other than methadone along with the research agenda at the HRB Centre for Primary Care Research in Ireland.

## Background

The HRB Centre for Primary Care Research http://www.hrbcentreprimarycare.ie aims to establish standards for the quality of care of vulnerable patient groups such as opiate users, with a particular emphasis on effective medicine management and the development of ICT interventions for use in General Practice (GP).

Drug misuse is a major public health problem, both nationally and internationally. There was an estimated 14,452 opiate users identified in Ireland in 2001 [1]. Drug misusers have an annual mortality rate six times higher than that for a general, age-matched population, with over two-thirds being due to drug overdoses [2].

Methadone is a synthetic opioid capable of blocking the euphoric effects of heroin and reduces cravings for the drug. Methadone therapy can be used as maintenance (methadone maintenance therapy or MMT), which involves the prescribing of methadone over an indefinite period of time. Alternatively, the dosage of methadone can be reduced over time (methadone reduction therapy) until the patient is drug free. MMT is the most common form of treatment. The aim of MMT is to replace illicit opiate use with a legally prescribed oral medication in order to provide the individual with a stable lifestyle. Evidence shows that effective MMT is associated with substantial health benefits for patients: decreasing use of heroin and other opioids; diminishing the transmission of opioid associated infectious disease such as hepatitis B and HIV; and reducing criminality behaviour in drug users who are stabilised on MMT [3-5].

Guidelines for treating opiate users in general practice are provided by the Irish College of General Practitioners (ICGP) [6]. The aim of these guidelines is to provide GPs with the relevant information and recommendations needed to treat opiate users. The guidelines, published in the form of an information booklet, cover a wide range of issues such as initial assessment, prescribing and dispensing methadone, urine screening, management options, and on-going monitoring and review to name but a few. However, the main problem is that these guidelines are not integrated into an electronic patient record, thus do not provide patient-specific recommendations for individual patients.

Methadone has been associated with drug related death, a Scottish study [7], reported that improper dosing and monitoring of patients taking methadone was associated with an increased risk of drug related death. In addition, co-prescribing of benzodiazepines with methadone has also been associated with drug related death. It is hypothesised that a clinical decision support system which provides guidance on drug prescribing (dose, duration, appropriateness), drug monitoring, and drug interactions may have a role in improving prescribing and so help to reduce the death rate in relation to prescribing factors. The HRB Centre aims to evaluate the use of a structured EHR and CDSS to improve MMT in general practice.

The move from paper based record keeping to structured electronic records for MMT will help information collection and analysis, auditing, policy development as well as decision making in Ireland. Irish GP systems do not have a purpose-built facility for recording methadone-related prescribing, supervision, and review. Although GPs are expected to comply with the clinical guidelines set out in a paper format, they are not provided with a corresponding computerised support for monitoring or drug prescribing as part of their routine data recording. Therefore a national audit of MMT in Ireland is by necessity manual and thus very costly. In addition, any attempt to evaluate the implementation of clinical guidelines into practice is currently very difficult, due to a reliance on paper based prescribing or, in the best case scenario, poor and unstructured notes in disparate systems. More importantly, safe prescribing and proper supervision management can not be guaranteed.

Addressing these issues is nontrivial. The aim of the project is to establish a system which facilitates data entry and decision support for GPs, as well as providing data collection and auditing for clinical authorities. The system is designed to be generic and flexible to incorporate the needs of data and knowledge management, with regards to medicine management in fields other than methadone prescribing, by meeting international standards and guidelines. This paper reports on our initial work on building an extensible and interoperable EHR and the potential for decision support within the devised framework.

### Overview of the open standards

The openEHR standard http://www.openEHR.org provides a complete open source software infrastructure for implementing an EHR in a clinical knowledge domain [8]. A key concept that is built into the openEHR architecture is the 2-level modeling paradigm which separates the technical reference model from the clinical knowledge itself. The reference model consists of generic data types and structures upon which the information system objects are built. The clinical knowledge is implemented using the archetype model and is completely separate and not implemented at all in the actual information system software itself.

Archetypes can be used to standardise the ever increasing clinical information structures and they offer a common gateway for exchange of data, being in a certain agreed form and of certain quality, across systems. Distinguishing constraints and clinical contents, archetypes have the advantage of separating responsibilities in knowledge management and sharing. Medical professionals can author archetypes. IT experts can build tools to automatically aggregate data from existing systems, the structures of which are constrained by the defined archetypes, into a common useful and extensible EHR model. Australia's Good Electronic Health Record (GEHR) framework [9], where the archetype concept originates, applied the approach to data from a hospital to GP communication project and a GP software integration project. They defined the mapping between source XML data formats and archetype fields, and employed an XSLT script to transform data from sources to a common EHR under the given archetype constraints. Another study investigated the expression of clinical data sets using archetypes and suggested archetypes provide meaningful, computer-processable knowledge and can help to improve the quality of the original data sets [10].

Archetypes define the structure of a series of fields with well-defined semantic relationships and a terminology defines the possible values for each field [11]. The terminology neutral archetypes can link to external terminologies, which gives archetype nodes unambiguous meaning via their references to specific terms of a given terminology. A good choice of terminology for binding is SNOMED CT [12], being the most comprehensive clinical terminology and comprising over 350,000 defined terms [13]. Ireland is currently not among the 9 Charter Members of International Health Terminology Standards Development Organisation (IHTSDO) which manages the international release of SNOMED CT. However, the HRB Centre has become an affiliate organisation and obtained an affiliate license for using SNOMED CT.

In the primary care setting, the International Classification of Primary Care (ICPC) [14,15] has been developed for GPs to code medical concepts. It has a biaxial structure and consists of 17 chapters based on body systems or problem areas and 7 components dealing with symptoms, diagnosis, treatment procedures and medication, and so on. The WHO has accepted ICPC2, including 1404 defined terms, as a classification for Primary Care. In Ireland, the national General Practice Information Technology (GPIT) group has the role of certifying GP Practice Software Management Systems. In its certification criteria, GPIT requires the use of a standard terminology model, e.g., ICPC2, ICD10, or SNOMED CT, for the semantic interoperation among the systems in electronic healthcare information exchange [16]. Vendors shall provide the ability to use ICPC2 to code elements of consultations and in addition either ICD10 or SNOMED as a more granular classification [17].

## Methods

### Design of an EHR for methadone treatment recording using open standards

Lawrence Weed proposed the now well-established method of Problem Oriented Medical Records (POMR) [18] and Subjective-Objective-Assessment-Plan (SOAP) [19]. They are designed to enhance the recording of consultations in medical practice and further support the logical thinking and analysis of the structured records. The approaches propose the following sections: 1) background information; 2) problem list; 3) progress notes (SOAP):

S: The *subjective *experience by the patient of the problem, or the "reason for encounter".

O: The *objective *clinical findings.

A: The *assessment *or diagnosis of the patient's problem.

P: The *process *of care or intervention *plan*.

The use of the problem-oriented approaches can clearly divide medical records into background and consultation episodes each being indexed by one or more problems. Information extraction or collection, and their analysis with regards to a particular problem will be relatively straightforward, since different problem parts are separately organised. Two major advantages of utilising this approach in building our EHR can be envisioned: 1) *Extensibility*. Opioid dependence is only one problem where methadone treatment is necessary. The independent arrangement of this part of the record can help its later merging into the record of other prescriptions addressing other problems, as well as its integration into other systems if the same model is adopted but where methadone treatment is not available. 2) *Decision support*. The continuous treatment on methadone is incrementally recorded in episodes of care. Being supplied with the knowledge of the past treatment history as well as the current condition of the patient, a decision support system can be designed and integrated into the EHR, to provide assistance in guiding the current consultation. This requires the computerisation of the clinical guidelines and applying them to the EHR, e.g. dosage and supervision recommendations over time, until the stabilisation of the patient on treatment.

We recognise that efforts have to be made in three directions, in applying the problem oriented approach to build the EHR and leverage its potential benefits of extensibility and decision support. Firstly, we must adopt *a standard to classify different problem areas*, so that record parts relevant to these can be distinguished, separately stored and easily queried. Secondly, we must adopt *an open standard for modelling methadone-related prescribing information *in the episodes of care without losing the generality or causing difficulties when later merging it into a larger data set. Thirdly, we must adopt *a terminology standard *for the codification of information pieces in the information model. In line with these requirements and the international standards outlined in Section 2, namely ICPC2, openEHR archetype, and SNOMED CT, we describe below a hierarchical EHR model of three levels, each being associated with a standard in the right position playing the right role.

#### Level 1: High level EHR model: care episodes and problem areas (standard: ICPC2)

**Figure 1:**
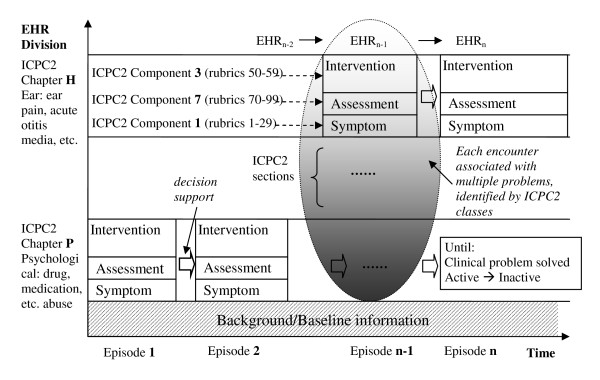
**A conceptual model of (de)composing EHR**.

The International Classification of Primary Care ICPC2 can classify all but the objective clinical findings (O) in the previously mentioned SOAP structure. The biaxial structure of ICPC2 has chapters which can model problem areas and components which can model symptoms, diagnosis and medication/intervention process. Figure 1 outlines a conceptual model for organising a complete EHR. The X axis represents recurring episodes over time, and the Y axis represents the problem areas separating the EHR. It combines the SOAP approach and the ICPC2 coding system but also includes a timeline factor as it plays a role in decision support. In an OpenEHR system compositions form the main content of the entire health record for a patient. Compositions can be viewed as units of information held in the EHR; each problem area represented on the Y axis can be modeled using these compositions e.g. a test result in ICPC2 chapter X (Event Composition) and a medication order in ICPC2 chapter Y (Persistent Composition).

Such a model marks clinical concepts in three dimensions of 1) episode number; 2) problem area numbers or ICPC2 chapters; 3) SOAP sections or ICPC2 components, and links them together on a continuing timeframe in a decision logic function. In addition, it may help to analyse co-morbidity and model drug interaction across problem areas.

In the context of dealing with an active ICPC2 chapter P in relation to methadone prescribing, such a model may be confronted with a limitation if not complemented with another system covering greater granularity than ICPC2, e.g. drug abuse is P19 but how to represent the methadone-specific concepts?

#### Level 2: Information model of EHR in the particular problem area of methadone treatment (standard: openEHR archetype)

The openEHR standard requires that an EHR system consists of at least an EHR repository, an archetype repository, terminology, and demographic information [8]. Demographics are included in the background information of Level 1 (and shown at the bottom of Figure 1) and the terminology will be covered by Level 3. Level 2 develops archetypes to confine the structures of EHR. Considering only methadone treatment, a band expanding along the X axis and falling into Chapter P on the Y axis in Figure 1 is our primary concern here. We need to represent all relevant components in a standardised manner, in order to support later data aggregation and interoperation.

The method advocated by openEHR for information recording, which it claims to be typical not just of clinical medicine but of science in general, models the iterative problem solving process as: *observations *→ *opinions/assessment *→ *instructions *→ *actions*. This is actually a synthesis model of Weed's problem-oriented method and roughly, one can map these from the block of three components shown in Figure 1: "*symptom*" becomes "*observation*", "*diagnosis*" becomes "*opinion or assessment*", and "*process*" becomes "*instruction*" and "*action*". The opinions/assessment part is where human hypothesis making or opinion forming bridges the incoming observations and outgoing instructions. It is also where the computer systems can support human decisions, integrating either evidence-based or guideline-based knowledge into the human personal knowledge base. From an opinion/assessment, further advice on prescribing instructions or dispensing actions can be suggested. In the entire process we mentioned above, the openEHR supports the modeling of information structures, by introducing equivalent kinds of archetypes: openEHR-EHR-*OBSERVATION*, openEHR-EHR-*EVALUATION*, openEHR-EHR-*INSTRUCTION*, openEHR-EHR-*ACTION*. Together with an "*ADMIN_ENTRY*", they form the five concrete subtypes of the "*ENTRY*" class (being a collection of "*Data Value*" and collectively forming "*Composition*"). All clinical information can ultimately be expressed in "Entries", or logical clinical statements. They are the most important in openEHR and make up the vast majority of archetypes defined for the EHR [8]. Some existing and reusable archetypes have been peer-reviewed and published in the openEHR online repository and are accessible at: http://www.openehr.org/knowledge/. We find they are general purpose, e.g. "medication order" cannot be taken directly but specialisation is needed for capturing the proper level of details. Nevertheless, such archetypes as alcohol consumption, tobacco use, or lab result may be included in a composition with our specifically designed archetypes, and support a larger variety of applications.

**Figure 2:**
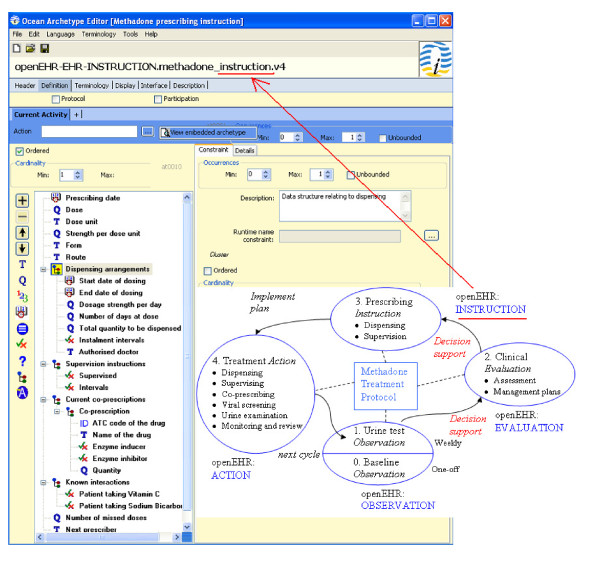
**Design of four types of archetypes (OBSERVATION, EVALUATION, INSTRUCTION, ACTION) for the episode of methadone treatment, with one example archetype of "Prescribing Instruction" being presented in an archetype editor**.

The archetype editor from Ocean Informatics was used for the design, creation and editing of the archetypes.Our design of archetypes for the methadone treatment protocol accurately reflects the minimum requirements imposed upon the information collection processes by the current practice and clinical guidelines but also incorporates additional fields that impact on the decision making. In the beginning, a patient claiming to be using illicit opiate approaches the GP. The current paper-based "*Addiction Assessment Form*" [6] is used to collect demographic data, drug, medical, and social history, and establish the presence and severity of opiate dependence, prior to commencing methadone treatment, e.g. abstinence may be achievable in patients with a short history of heroin use and therefore methadone treatment not required. The form structure guides the construction of a baseline archetype. From this initial *observation (0) *and the following *observation (1) *of urine screening results on a regular basis (together forming the starting node of the circle shown in Figure 2), the GP establishes knowledge about the patient's heroin habit and forms current management plans in an *evaluation (2) *step. Based on this, the prescribing of methadone including dosage and supervision arrangement will be given in an *instruction (3)*. The paper-based "*Methadone Prescription Form*" [6] is used to construct the core of this archetype. Since methadone is metabolised by the liver, drugs which induce liver enzymes may reduce the clinical effects and similarly enzyme inhibitors may potentiate the effects. Thus co-prescribing information and other pharmacodynamic interactions become complementary elements, shown in the archetype's tree structure of Figure 2, as they influence the correct dosage prescribed. Instructions can be taken into *action (4)*, by the patients or care providers or labs, e.g. viral screening or urine examination. The two kinds of paper-based forms are not the only sources from which we elicit information to build archetypes. Annual audit is provided by ICGP/HSE appointed audit nurses and the care provided by GPs to their methadone patients is reviewed and referenced to the current guidelines. In the "*Audit Criteria*" table [6], items include virology assessment, urinalysis, and other psychoactive drugs prescribed. Since the treatment of opiate users aims to improve the overall physical, psychological and social health of individuals, ongoing review of the treatment plan is essential to determine how effective it is in relation to patient's illicit or non-prescribed drug use, as well as their health risks associated with illicit drug use, particularly the risk of HIV, hepatitis A, B, and C. Thus, an archetype for monitoring and review becomes a necessary addition. The above archetypes designed for the methadone treatment protocol are being submitted to the Clinical Review Board of openEHR for review so that they will become reusable and enable interoperation. Now imagine when a patient comes to the practice and a new episode of care is established, the EHR expands along the X axis and within the Chapter P band on the Y axis of Figure 1. Information is gathered step by step and validated against the defined archetypes around the cycle shown in the bottom right part of Figure 2. Eventually, an extensible and interoperable EHR can be built up incrementally.

#### Level 3: Archetype linkage to a terminology (standard: SNOMED CT)

**Figure 3:**
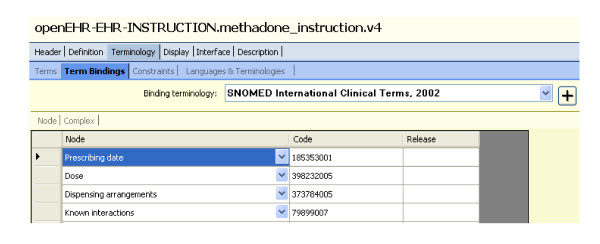
**Binding of the terms in our archetype to SNOMED CT**.

Level 1 gives high level division on problem areas and Level 2 defines the information structures falling into a given problem area that one can use for applications. In the following level, the fields in such information structures need to be mutually understandable, and their semantic meanings agreed by the wide community, when a party downloads an archetype to validate an imported data structure from another party. The concept of methadone, as a substance of opioid treatment is certainly associated with ICPC2's P Chapter and information structures around their prescribing can be defined for various applications. ICPC2 or archetypes cannot uniquely identify the term methadone therefore a linkage terminology is required for this purpose. SNOMED CT, has a detailed conceptualisation of clinical terms, e.g. methadone (substance) has a unique concept ID of "387286002" and "*is-a*" "drugs used to treat addiction (substance)". Figure 3 shows the binding of terms from our example "Prescribing Instruction" archetype to SNOMED.

**Figure 4:**
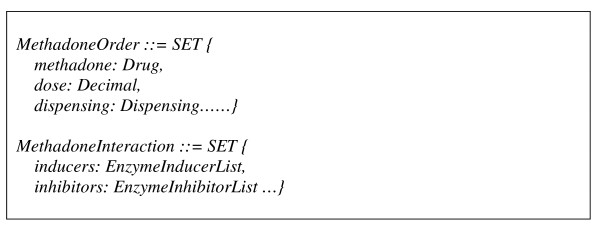
**Scheme of concept 'drug'**.

The greater power of binding sharable and reusable archetypes and a community-agreed standard terminology is magnified in concept relationship reasoning where both archetypes and terms are referenced and their meanings computable in decision logics, within or across applications. It has been argued in [11] that terminology provides the ability to do hierarchical inference on values in instances of data created in accordance with the information model. Similarly to an example given in their testimony, an information model for methadone prescribing order or instruction could be concisely expressed as below. Also given is a structure (see scheme on Figure 4) for expressing the interaction between methadone and enzyme inducers or inhibitors which decreases or increases the clinical effect of methadone and thus requires a corresponding methadone dosage change.

**Figure 5:**
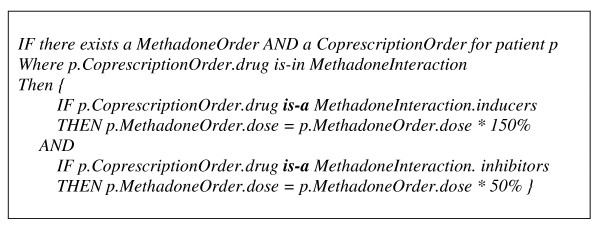
**Scheme of concept 'Methadone Interaction'**.

In the above scheme, "*Drug*" is a concept whose semantic meaning has been agreed and sub-categories of this concept can be related in a terminology. Assume an information model for co-prescription order exists, similar to that for methadone, a decision logic for adjusting methadone dosage is shown below, where "*is-a" *is used, specifically in this context, to judge if a drug belongs to an enzyme inducer or inhibitor. However, in the presence of a semantic network of drugs organised in a classification hierarchy (effectively many generically defined and globally validated "*is-a*" among every possible drug types), the inference of a drug as being an inducer or inhibitor via the network will eventually diminish the need of listing/identifying all such categorisation, e.g. inducers or inhibitors in the "*MethadoneInteraction*" structure (sees scheme on Figure 5), for every single application.

### Implementation of the interface and the guideline-directed decision support

#### The data entry interface

**Figure 6:**
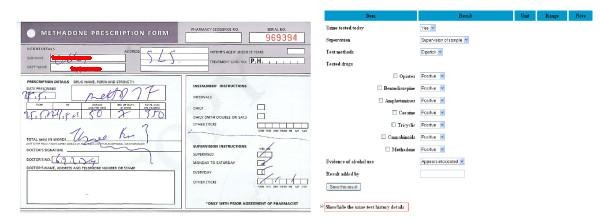
**Anonymised paper-based methadone prescription form *(left) *and the corresponding computerised user interface *(right)***.

The interface was developed using jdk1.6, Java Server Pages 1.1 and Apache Tomcat v6.0 Server. The database layer was developed using MySql Server 5.1 Database.We have implemented data entry interface for: *A) Baseline Information; B) Urine Testing; C) Methadone Dosage and Dispensing Arrangement; and D) Monitoring/Review*. The structure of data for collection matches that of the defined archetypes in Section 3 and can be coded accordingly. Figure 6 shows on the left hand side, a paper-based prescription form used in the current practice and on the right hand side, part of Section C of our interface. The structured data entry interface only collects the very essential information and not all archetype fields defined in Figure 2 have associated collection points in the interface, e.g. "prescribing date" can be populated into the actual EHR automatically by the programme; "missed doses" can be calculated from the record history in EHR; 1 mg/1ml formulation of liquid methadone by oral consumption is fixed in the Irish setting, despite the definition of "strength", "form", and "route" in the archetype for interoperation purpose.

Initially the interface was designed as a series of questions and answers. Following interviews with a GP who prescribes methadone the initial design was found to be too long to use in regular consultations. Considering the standard paper-based forms, the data entry template used in their surgery, and additional features relevant to decision support, we adapted the previous interface in terms of the structure and made the data entry as concise and clear as possible. Finally, one web page has been deployed for collecting the one-off baseline information (A), one for the urine testing results (B), one for the weekly prescribing information (C), and the last one for the six-monthly review (D). The core prescribing or Section C is the only mandatory data entry at each appointment. The GP will be requested to carry out urine testing only if such tests have been ignored or requests for testing have been overridden for a sufficient number of times. Each page is accommodated in a single screen with option selection questions, which are straightforward to go through and answer. This will be fitted and rendered more GP-friendly. Feedback on the interface design and usability was initiated from potential GP users and existing clinical decision support system designers and lecturers. Such enhancements include: listing previously recorded urine testing or prescribing data below the main data collection section with a show/hide switch option as highlighted in Figure 4; for the current prescribing, suggesting the options of 3 most recently prescribed doses beside a new dose field for filling in; automatic floating layer popup when the mouse is placed over a term, providing further explanation or clarification when necessary.

#### Structuring guidelines

**Figure 7:**
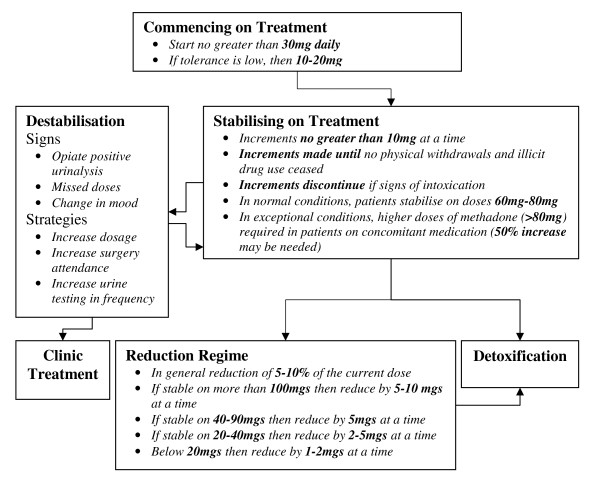
**The recommended methadone treatment protocol structured as a decision-making flowchart (care pathway)**.

A successful integration of the EHR and CDSS relies on the ability to generate recommendations for the current prescribing based on previous records as per the guidelines. The current ICGP guidelines on methadone treatment are well organised into various management sections in their published book, depending upon the status of the patient. The first stage in designing a system to implement electronic guidelines was to represent the knowledge of the guidelines as a decision making flowchart in Figure 7. Each node represents a status where a drug user may transit from one to another, and each node consists of the prescribing recommendations associated with that status.

#### Clinical decision support

We have shown on Figure 2 that decision support can facilitate the move from observation to evaluation and then to prescribing instruction and action, during the transition among various stages of methadone treatment.

Experienced level-two prescribing doctors usually rely on pattern recognition, or the previous knowledge of treating patients with similar issues. Doctors at level-one in methadone prescribing may need frequent reference to the guidelines for assistance. Automatic decision support could potentially provide valuable integrated support to the clinical process of methadone prescribing and associated treatment of these patients. The envisioned decision support component will take the current patient EHR, judge their status (nodes such as commencement, stabilising, reduction, etc. in Figure 7), apply the guidelines associated with the status upon the past prescribing history (rules under the node titles in Figure 5), and generate recommendations for the current prescribing for that patient. This decision support could be used to explicitly give recommended dosage and supervision arrangement when a GP commences a new consultation with a patient. Alternatively, the decision support could alter/remind if the given prescription does not comply with the guidelines and an alternative dosage or supervision arrangement is thus suggested for consideration. The other useful functions that decision support can provide include the monitoring of the patient's status change and reminding or alerting the GP to consider adjusting management plan, e.g. reminding them of the possibility of carrying out a reduction regime when a patient has reached stabilisation for a certain period of time or automatically alerting the destabilisation if certain doses have been missed. We believe such a model of decision support, providing recommendations rather than assessments, at the time and location of decision making, automatically as part of the clinical workflow, meet the criteria of a decision support system's ability to improve clinical practice [20].

We are investigating the use of a standard knowledge representation language for the documentation of clinical guidelines (management options, flowcharts, rules, etc.). A common template will be designed for the management of knowledge (not limited to methadone prescribing) in a standard computerised format. The represented guideline knowledge will be able to be queried, retrieved, triggered, and applied by a knowledge engine at runtime. Such an engine, effectively aggregating EHR episodes, and operate upon information structures constrained by the well-defined archetypes. As an example, Figure 5 illustrates the guidelines in relation to dosage value change over transition, which represents one field in the "Prescribing Instruction" archetype.

## Results

A difficulty we identified in the beginning of our study is the lack of enthusiasm among various GP software vendors in relation to the integration of a methadone treatment package into existing systems. Therefore, the best sustainable solution is to develop an EHR that adopts international standards and supports prescribing as per the paper guidelines. This will enable the later interoperation among systems and support linkage from various existing records to the methadone-related records. Also, the EHR needs to be extensible to cover medicine management in general as per the Centre's programme and not limit itself to methadone.

During the initial development process, well-defined archetypes were designed which guided the development and structure of the database schema and interface layers, reducing mapping issues at a later stage of development. The use of archetypes captures the ever changing requirements in the healthcare domain and externalises them in constrained data structures. With runtime mapping and interpretation, it is possible to do dynamic data aggregation and knowledge understanding without changing the underpinning software, as evidenced in our previous work first on software adaptivity [21] and later security modelling for healthcare information systems [22], both in distributed environments. Defining archetypes explicitly rather than having all information structures hard-coded, we have our methadone treatment EHR system open for data aggregation and sharing: we publish our archetypes, send the data to whoever is authorised to receive it, they download the published archetypes, use them to validate data, understand data structures and contents, query the structures, retrieve the required information, and import them to their own systems for benefits. The relative immaturity of the OpenEHR architecture is the main limitation of the approach used in this study, however the model was found to be sufficiently descriptive to represent the clinical data. An alternative approach would be to use HL7 V3 Clinical Document Architecture (CDA) which has similarities in that it supports template design however CDA would only be suitable to model extracts but not theentire EHR.

We are liaising with the Irish College of General Practitioners (ICGP), which has authorised governance over the methadone prescribing guidelines, to promote the creation of a more useable and integrated software [23]. We will evaluate our system using a clustered randomised controlled trial (RCT), enabling a robust evaluation of the impact of ICT intervention in terms of patient retention in treatment. Retaining patients in MMT is essential as patients who leave voluntarily or have been expelled have increased mortality risks [24,25]. Patients are most likely to leave MMT as they destabilise on treatment. We anticipate that our system will flag a warning to the treating GP when a patient is showing signs of destabilisation, providing them with evidence based strategies to re-engage the patient, thus retaining and stabilising the patient and reducing the likelihood of mortality. Our system will also be evaluated in terms of the process of care, and patient outcome measures, at 12 months follow up. By demonstrating the potential benefits to the ICGP and the wider community, we hope that the GP software vendors will be urged to integrate the decision support component into their systems and enable its promotion in Ireland.

## Discussion and Conclusions

This paper describes a concept model/approach for the use of three international standards of ICPC2, openEHR, and SNOMED CT to build a hierarchical EHR model and demonstrates its application to the recording of methadone treatment. Such an approach contributes to the ordinary EHR extensibility, interoperability, and decision support. Separately built systems can have data stored in their individual databases and information exchanged and understood unambiguously across systems, structurally via archetype constraints and conceptually via SNOMED CT. In using ICPC2, we can aggregate data from various clinical sources, based on the same symptom codes on a particular clinical area and conduct studies on the analysis of the outcomes of various processes commissioned for the same symptoms. Related to this, such a paradigm can also enable the accumulation of evidence from the actual use in practice with regards to adherence to guidelines and associate them with outcomes. In this way, it can suggest improvements and updates to the guidelines based on evidence.

From the viewpoint of further perspectives, the ultimate aim of building such a structured EHR is the development of a fully integrated CDSS, reducing prescribing errors and supporting auditing, as hand-written prescriptions are required for controlled drugs in this country. In our future work, we will develop a knowledge interpretation engine and use a standard knowledge representation to capture the guidelines in a computable form. We will also investigate the dynamic generation of data collection use interfaces from archetypes, which is technically feasible and indeed what archetypes should support, data entry fields corresponding to their archetype structure counterparts and linking to SNOMED terms and these being recorded in an EHR of the same structure.

Last but not least, when the full decision support is developed, it will present a one-page printable summary of the consultation upon the confirmation of the prescribing, including the last screening, co-prescribed drugs, methadone prescribed, any change since the past consultation, and the situations about missed appointments, urine test opioid positive/negative, suspected alcohol use - all associated with the status monitoring on whether stabilising or destabilising. Also, if appropriate, it will suggest any alternative to the dosage or supervision level for the current consultation.

## Abbreviations

CDA: Clinical Document Architecture; CDSS: Clinical Decision Support System; HER: Electronic Health Record; GEHR: Good Electronic Health Record; GP: General Practice; GPIT: General Practice Information Technology; HIV: Human immunodeficiency virus; HRB: Health Research Board; ICD10: The International Statistical Classification of Diseases 10th Revision; ICGP: Irish College of General Practitioners; ICPC2: International Classification of Primary Care; ICT: Information and communications technology; IHTSDO: International Health Terminology Standards Development Organisation; MMT: Methadone Maintenance Therapy; SNOMED CT: Systematized Nomenclature of Medicine - Clinical Terms; SOAP: Subjective-Objective-Assessment-Plan; POMR: Problem Oriented Medical Records; XML: Extensible Markup Language; XSLT: Extensible Stylesheet Language Transformations;

## Competing interests

The authors declare that they have no competing interests.

## Authors' contributions

All authors have made substantial contributions to the paper; they have been involved in drafting the manuscript and revising it critically for important intellectual content. All authors have given final approval of the version to be published.

## Pre-publication history

The pre-publication history for this paper can be accessed here:

http://www.biomedcentral.com/1472-6947/11/5/prepub
